# Regulation of Cell Signaling Pathways and miRNAs by Resveratrol in Different Cancers

**DOI:** 10.3390/ijms19030652

**Published:** 2018-02-26

**Authors:** Ammad Ahmad Farooqi, Sumbul Khalid, Aamir Ahmad

**Affiliations:** 1 Institute of Biomedical and Genetic Engineering (IBGE), Islamabad 44000, Pakistan; ammadfarooqi@rlmclahore.com; 2 Department of Bioinformatics and Biotechnology, International Islamic University, Islamabad 44000, Pakistan; sumbul.khalid@iiu.edu.pk; 3 Department of Oncologic Sciences, Mitchell Cancer Institute, University of South Alabama, Mobile, AL 36604, USA

**Keywords:** resveratrol, cell signaling, miRNAs

## Abstract

Genomic and proteomic studies have helped improve our understanding of the underlying mechanism(s) of cancer development and progression. Mutations, overexpressed oncogenes, inactivated/downregulated tumor suppressors, loss of apoptosis, and dysregulated signal transduction cascades are some of the well-studied areas of research. Resveratrol has gained considerable attention in the last two decades because of its pleiotropic anticancer activities. In this review, we have summarized the regulation of WNT, SHH (sonic hedgehog)/GLI (glioma-associated oncogene homolog), TGFβ1 (transforming growth factor beta 1)/SMAD, NOTCH, TRAIL (tumor necrosis factor-related apoptosis-inducing ligand), STAT (signal transducer and activator of transcription), and microRNAs by resveratrol in different cancers. The importance of these signaling pathways in cancer progression, along with their modulation by resveratrol, is discussed. Further, we also evaluate the mechanisms and implications of the downregulation of oncogenic miRNAs and the upregulation of tumor suppressor miRNAs by resveratrol, both of which also define its ability to inhibit tumor growth and metastasis. It is envisioned that designing effective clinical trials will be helpful for the identification of resveratrol responders and non-responders and the elucidation of how this phytochemical can be combined with current therapeutic options to improve their clinical efficacy and reduce off-target effects.

## 1. Introduction

Using high-throughput technologies, it has been shown that the bulk tumor includes a diversified collection of cells that harbor characteristically unique molecular features with variable levels of sensitivity to therapeutic options [1,2]. This heterogeneity results in an uneven distribution of genetically distinct subpopulations of tumor cells across and within disease sites (spatial heterogeneity) [3,4]. In addition, tumors are marked by temporal heterogeneity, wherein cells temporally change their molecular makeup [3,4]. Combining high-throughput genotyping platforms and next-generation sequencing technologies has brought us one step closer in the pursuit for genetic variants, from SNPs (single nucleotide polymorphisms) to large-scale copy number variants. Translational and functional studies have demystified the underlying mechanisms and bio-molecular signatures of difficult-to-treat cancers. Natural product chemistry and the discovery of drugs from plants (phytochemicals) has enjoyed a renaissance in the past decade [5,6]. Based on the insights gleaned from decades of research, it seems clear that off-target effects of modern medicines and rapidly developing resistance against different therapeutics are some of the challenges that must be overcome. Much attention is currently being given to the identification of natural products that can modulate multiple targets, are significantly effective, and minimize off-target effects [6,7]. In accordance with this approach, there is a rapidly growing list of natural products with considerable medicinal importance and substantial pharmacological properties [8]. 

Resveratrol, a polyphenolic phytoalexin, is an extensively studied phytochemical with remarkable pharmacological properties and a unique ability to modulate multiple targets in different cancers [9]. It regulates oxidative stress, which is an interesting aspect of its anticancer activity [10,11]. Resveratrol has a molecular weight of 228.247 g/mol and a topological polar surface area of 60.7 A^2^ [12]. Because of the rapidly accumulating wealth of information about resveratrol, many review papers have summarized important biological mechanisms targeted by resveratrol for the treatment of different cancers [13,14,15,16]. Most recently published reviews have provided a summary of the role of resveratrol in different cancers; however, in recent years, none of the reviews have provided an in-depth and comprehensive overview of the cell signaling pathways targeted by resveratrol in different cancers [17,18,19]. We have exclusively focused on various signal transduction cascades and the miRNAs that are modulated by resveratrol in different cancers. In particular, in this review, we have summarized the regulation of TGFβ1/SMAD, WNT, SHH/GLI, NOTCH, TRAIL, STAT pathways, and the oncogenic as well as tumor suppressor miRNAs, by resveratrol.

## 2. Regulation of the TGFβ1/SMAD Pathway

**Figure 1:**
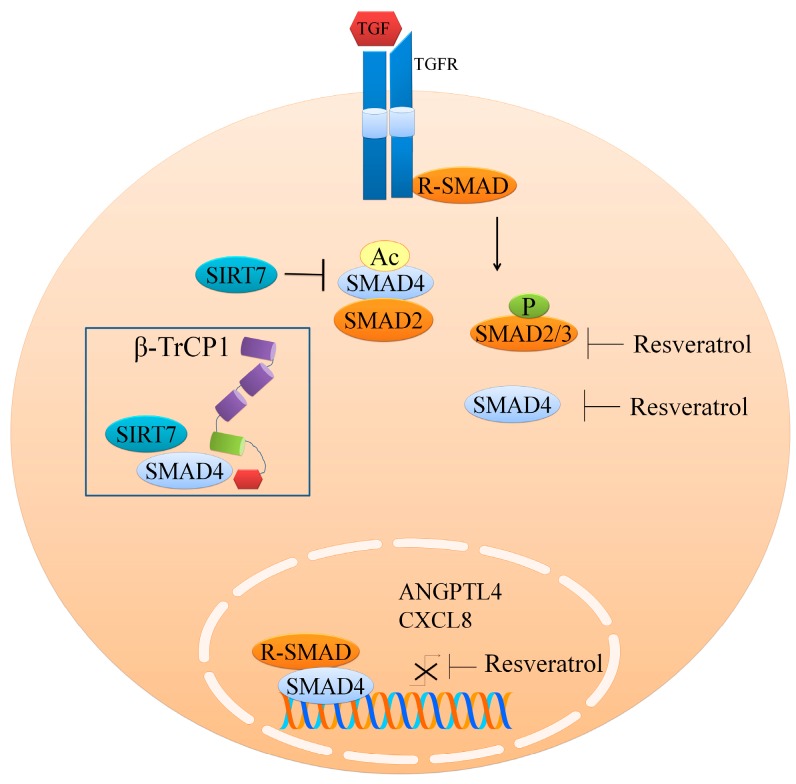
TGF/TGFR signaling axis. SMAD proteins play a central role in transducing the signals intracellularly. SIRT7 modulates SMAD4 degradation via β-transducin repeat-containing protein (β-TrCP1). SMAD4, β-TrCP1, and SIRT7 complex formation results in shutdown of the TGF pathway. SMAD: Sma Mothers Against Decapentaplegic; β-TrCP: β-transducin repeat-containing protein; CXCL8: chemokine CXC motif, ligand 8; TGF: transforming growth factor; ANGPTL4: angiopoietin-like 4.

TGFβ1/SMAD pathway is well known to contribute to cancer development and progression (Figure 1). Signalosome formed by phosphorylated receptor-mediated SMADs triggers the accumulation of active SMAD complexes in the nucleus, which transcriptionally upregulate target genes in conjunction with transcriptional factors, histone modifiers, and chromatin-remodeling machinery [20]. In this section, we will provide an overview of the mode of action of resveratrol to inhibit SMAD-mediated intracellular signaling. Resveratrol effectively inhibits SMAD proteins by either affecting post-translational modifications by different enzymes to target SMAD proteins for degradation or by inhibiting the phosphorylation and activation of SMADs. 

In a study demonstrating regulation of the TGFβ1/SMAD pathway by resveratrol, lung metastases were developed in Balb/c mice by intravenous injection of highly metastatic murine 4T1 cells [21]. Further, intravenous injection of the SIRT7-inhibited development of these metastatic nodules and SIRT7-knockdown induced activation of the TGF-β pathway in 4T1 and MDA-MB-231 cells, while SIRT7 overexpression exerted inhibitory effects on the TGF-β pathway. SIRT7 physically interacted with and de-acetylated SMAD4 [21]. Significantly increased levels of acetylated lysine residues of SMAD4 were noted in SIRT7-silenced cancer cells. The binding affinity of SMAD4 for SMAD2 markedly decreased in cells that ectopically expressed SIRT7. When protein synthesis was inhibited by cycloheximide, ectopically expressed SIRT7 promoted SMAD4 degradation, and SIRT7 inhibition protected SMAD4 from degradation machinery [21]. Furthermore, K428R, which mimicked a hypo-acetylated form of SMAD4, accelerated its degradation, whereas K428Q, which resembled a hyper-acetylated form of SMAD4, inhibited its degradation. SIRT7 deletion by CRISPR/Cas9 inhibited HA-SMAD4 ubiquitination, whereas SIRT7 overexpression increased ubiquitination. SIRT7 modulated SMAD4 degradation by β-transducin repeat-containing protein (β-TrCP1). Protein studies have provided evidence of the interaction of SMAD4, β-TrCP1, and SIRT7 [21] (Figure 1). β-TrCP1 inhibition abolished the increased ubiquitination and accelerated SMAD4 degradation caused by ectopically expressed SIRT7. Resveratrol dose-dependently enhanced deacetylation of HA-SMAD4. It also exerted repressive effects on TGF-β1-induced mesenchymal morphology, downregulated SMAD4, and suppressed cellular migration. TGF-β responsive genes *ANGPTL4* and *CXCL8*, required for breast cancer lung metastases and the development of resistance against drugs, were considerably repressed by resveratrol [21].

Resveratrol has been reported to reduce phosphorylated-SMAD2/3 in colorectal cancer LoVo cells [22]. Resveratrol (150 mg/Kg) reduced metastatic lesions in the lungs and liver of mice orthotopically transplanted with LoVo cells. In alveolar rhabdomyosarcoma PLA-802 cells, resveratrol (40 μmol/L) downregulated SMAD4 [23]. Upon treatment with resveratrol (20 μmol/L), no significant changes in expression of TGF-β1 and SMAD4 were noticed; however, at a dose of 40 μmol/L, resveratrol significantly decreased the expression of SMAD4 and TGF-β1 [23]. Resveratrol decreased expression of TGFβ-2 in SCCs and skin of p53^+/−^/SKH-1 mice chronically irradiated with UVB [24]. There was a 2–3-fold increase in the invasive capacity of cells that expressed constitutively active Akt. Overexpression of active Akt induced TGF-β2 in the absence of resveratrol, which suggested a clear association between TGFβ2 and Akt. Active Akt significantly impaired resveratrol-induced inhibitory effects on TGF-β2. Resveratrol dose-dependently reduced phospho-SMAD2/3 levels and completely abolished the phosphorylation of SMAD2/3 at a dose of 100 μM [24].

These findings indicate that resveratrol affects the TGFβ1/SMAD pathway via post-translational modifications, either by degrading SMADs or inhibiting their activity by blocking the phosphorylation of SMADs.

## 3. Regulation of the WNT Pathway

**Figure 2:**
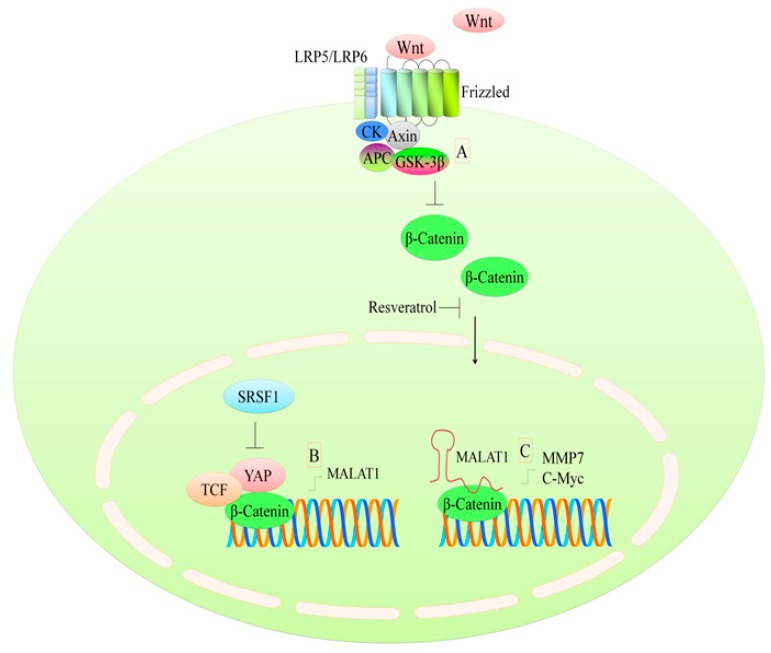
(**A**) WNT/β-catenin signaling axis. Resveratrol effectively inhibits the nuclear accumulation of β-catenin. β-catenin works synchronously with various proteins to modulate the transcription of different genes; (**B**) SRSF1 inhibits YAP and represses the β-catenin-mediated upregulation of MALAT1; (**C**) β-catenin works with MALAT1 and increases the expression of MMP7 and C-Myc. APC: adenomatous polyposis coli; CK: casein kinase; GSK-3β: glycogen synthase kinase 3 beta; LRP: low-density lipoprotein-receptor-related protein; MALAT1: metastasis-associated lung adenocarcinoma transcript 1; MMP&: matrix metalloproteinase-7; SRSF1: serine and arginine rich splicing factor 1; TCF: T-cell factor; YAP: YES-associated protein; 65-KD.

A WNT ligand is a secreted glycoprotein that binds to Frizzled receptors and results in the formation of a larger cell surface complex with low-density lipoprotein-receptor-related protein (LRP5/6) [25] (Figure 2). In the absence of any signal, β-catenin is post-translationally modified by the APC/AXIN/GSK-3β-complex leading to its ubiquitination and degradation by proteasomal machinery. When the WNT pathway is active, β-catenin gets sequestered away from degradation machinery and moves into the nucleus to trigger the expression of target genes. There has been a focus on strategies to inhibit the transportation of β-catenin into the nucleus [25,26].

Resveratrol can downregulate WNT2 and upregulate AXIN2 in Colo16 cells [27]. Furthermore, there was an earlier induction of apoptosis in resveratrol-treated β-catenin-silenced Colo16 cells [27]. Resveratrol dose-dependently decreased exogenously overexpressed Myc-tagged TCF4 protein in colorectal cancer cells [28] and increased TCF4 phosphorylation through ERK- and p38-MAPK-modulated pathways. Knockdown of TCF4 reduced TCF/β-catenin-regulated transcriptional activities and sensitized cancer cells to resveratrol-mediated apoptosis [28].

Response to resveratrol was noted to be highly heterogeneous among glioma stem cells. Resveratrol inhibited cell proliferation, increased cell mortality, and strongly reduced the motility of the cells [29]. In GBM2, G166, GBM7, and G179 cell lines, both WNT1 and MYC were upregulated, while in G144 cells these two genes were downregulated by resveratrol. FZD4 and TCF7 changed only in 3 out of 7 GSC lines after resveratrol treatment [29]. Resveratrol significantly reduced cyclin D1 and β-catenin in xenograft breast tumors [30]. β-catenin overexpression markedly reduced resveratrol-mediated cytotoxic effects, which indicated that resveratrol inhibited breast cancer stem-like cells and induced autophagy, at least partially, by suppression of the Wnt/β-catenin cascade [30]. In HCT116 cells, resveratrol reduced β-catenin [31]. Resveratrol did not appear to influence the total levels of GSK-3β but did decrease GSK-3β phosphorylation. A β-catenin/Tcf4 reporter assay provided evidence that resveratrol, concentration-dependently, inhibited reporter activity [31]. 

β-catenin has been shown to interact with different non-coding RNAs and transcriptionally upregulate their expression, thereby stimulating the expression of a target gene network. Resveratrol increases the cytosolic levels of β-catenin with a concomitant inhibition of its nuclear accumulation (Figure 2), with little effects on the total cellular β-catenin [32]. Resveratrol exerted inhibitory effects on the Wnt/β-catenin pathway through the regulation of MALAT1. The use of lithium chloride inhibited GSK3β from binding to β-catenin and triggered Wnt/β-catenin pathway activation. Resveratrol dose-dependently downregulated long non-coding RNA-MALAT1 [32]. Knockdown of MALAT1 impaired the nuclear accumulation of β-catenin, which resulted in the transcriptional repression of MMP7 and c-Myc, while MALAT1 overexpression promoted the transcriptional upregulation of MMP7 and c-Myc (Figure 2). The data clearly suggest that MALAT1 and β-catenin co-existed in the nucleus, and the targeted inhibition of MALAT1 is necessary to inhibit synchronous activity of MALAT1 and β-catenin for the transcriptional repression of target genes [32]. YES-associated protein (YAP) upregulated MALAT1 transcriptional and post-transcriptional levels, whereas serine/arginine-rich splicing factor 1 (SRSF1) resisted YAP-mediated effects [33]. SRSF1 exerted inhibitory effects on YAP activity and prevented its co-existence with TCF/β-catenin on the promoter region of MALAT1 [33] (Figure 2). These two reports together are helpful in putting different pieces of an incomplete jigsaw puzzle together. It seems clear that β-catenin co-operates with different proteins to trigger the expression of MALAT1 and works synchronously with MALAT1 to drive the expression of WNT signaling target genes.

Despite these studies on the regulation of the WNT pathway by resveratrol, there remain a few outstanding questions that need to be adequately addressed to understand mechanistic details. For example, the modulation of LRP5/6 and SFRPs by resveratrol has not been sufficiently studied. Future studies need to comprehensively focus on the detailed mechanism(s) of regulation of WNT signaling by resveratrol so that the information can be utilized for the targeting of WNT signaling by resveratrol in clinical settings.

## 4. Regulation of the SHH/GLI Pathway

Inappropriate activation of the Sonic hedgehog–GLI signaling cascade has been extensively studied in different cancers [34,35,36]. BCR-ABL is an oncoprotein that re-wires intracellular signaling cascades in chronic myeloid leukemia and Philadelphia chromosome-positive acute lymphoblastic leukemia [37]. In vitro and preclinical studies have shown that imatinib exerted preferential inhibitory effects on the viability and expression of BCR-ABL in imatinib-sensitive K562 cells, but imatinib was not effective against K562R cells that overexpressed BCR-ABL. Both K562R (imatinib-resistant) and parental K562 cells expressed mRNAs of molecules involved in the SHH pathway, including SHH, Smoothened (SMO), Patched (PTCH), and GLI-1. There was a significant reduction in BCR-ABL in GLI-1-silenced K562R and K562 cells. The use of SHH peptide not only enhanced GLI-1 and SHH but also upregulated BCR-ABL in tested cell lines. Resveratrol effectively inhibited SMO and BCR-ABL and exerted inhibitory effects on the nuclear accumulation of GLI proteins to transcriptionally upregulate the expression of cancer promoting genes [37]. PTCH, SMO, and GLI-1 were also inhibited in resveratrol-treated colorectal cancer HCT116 cells [38]. Resveratrol inhibited the nuclear accumulation of GLI-1 in interleukin-6 (IL-6)-stimulated HL-60 cells [39].

Resveratrol and cyclopamine have been reported to synergistically inhibit metastases and reduce the invasive potential of gastric SGC-7901 cancer cells [40]. Resveratrol was noted to inhibit GLI-1 activity and downregulate Snail and *N*-cadherin in SGC-7901 cells. Previously, it has been reported that Snail repressed the expression of E-cadherin, but resveratrol upregulated E-cadherin and markedly reduced the metastasizing potential of SGC-7901 cells [40]. Based on the reports discussed above, resveratrol seems to significantly affects the SHH-Gli pathway.

It is now well established that CpG islands are heavily hypomethylated in cancer cells [41]. Resveratrol enhances the methylation levels of CpG sites within enhancers of MAML2 and GLI2. Interestingly, active enhancers are enriched with H3K27ac. Data obtained through the chromatin-immunoprecipitation technique provided information about the presence of histone marks at MAML2 in cancer cells but its occupancy reduced considerably in resveratrol-treated cancer cells [41]. Occupancy of activating histone marks (H3K9acetylation) and repressive histone marks (H3K27tri-methylation) was biochemically analyzed. Data suggested a 30% decrease in H3K9ac and a 2-fold increase in H3K27me3 occupancy within the MAML2 enhancer, whereas resveratrol induced an increase in DNA methylation levels. OCT1, a transcription factor, transcriptionally upregulated MAML2; however, resveratrol inhibited the binding of OCT1 to the MAML2 enhancer. Resveratrol with a concentration of 15 μM promoted the accumulation of DNMT3B in the MAML2 enhancer in MCF10CA1a cells, resulting in epigenetic inactivation [41].

## 5. Regulation of the NOTCH Pathway: A Double-Edged Sword

The NOTCH pathway is involved in the regulation of different stages of cancer [42,43]. Although some reports document efficient inhibition of different proteins of the NOTCH pathway by resveratrol to inhibit cancer, there are conflicting reports that resveratrol can activate the NOTCH pathway, leading to its anticancer activity. Resveratrol has been noted to induce differentiation-associated genes in anaplastic thyroid carcinoma mainly through Notch1 activation [44]. Resveratrol was a strong inducer of TTF1, which increased 4-fold at 25 μmol/L and 6-fold at 50 μmol/L in tested cell lines. Significantly elevated TTF2 was observed at 50 μmol/L in thyroid cancer cells. Moreover, there was an increase (5-fold) in Pax8 in HTh7 cells at a 50 μmol/L dose of resveratrol. The upregulation of Pax8, TTF1, and TTF2 was observed in resveratrol-treated NOTCH1-competent 8505C cells. However, no detectable rise has been found in the levels of Pax8 or TTF1 in resveratrol-treated, NOTCH1-silenced ATC cells. Tumor volume decreased considerably (~69%) after five injections of resveratrol in mice xenografted with either HTh7 or 8505C cells [44]. 

Resveratrol (50 µM) increased both active p53 and NOTCH-1 in glioblastoma cells [45]. Western blot data suggest that expression of the Notch-1 intracellular domain (NICD) was 3.2-fold and 2.9-fold higher in the A172 and T98G cells, respectively, after resveratrol treatment. Similar patterns were observed and p53 was also found to be upregulated as evidenced by 1.2-fold and 1.1-fold higher expressions in A172 and T98G cells, respectively, after treatment with resveratrol [45]. Taken together, the effect of resveratrol on NOTCH signaling seems to be context-dependent. When NOTCH signaling is oncogenic, it is effectively inhibited by resveratrol; however, when it leans towards a tumor-suppressive action, it is potentiated by resveratrol.

## 6. Regulation of TRAIL Signaling

The discovery of TRAIL revolutionized the field of molecular oncology because of its ability to differentially target cancer cells while leaving normal cells intact [46]. However, circumstantial evidence has also indicated the development of resistance against TRAIL-based therapeutics. TRAIL resistance emerged as a major setback in the standardization of therapy, and attempts have been made to sensitize resistant cancer cells to TRAIL. Analyses of different cancers have shown an imbalance in stoichiometric ratios of pro- and anti-apoptotic proteins. However, over the past 10 years, there has been a worldwide resurgence of studies emphasizing different strategies for restoring TRAIL-induced apoptosis in resistant cancer cells. Resveratrol has shown potential as a TRAIL sensitizer.

Resveratrol has been found to increase the cell-surface expression of NKG2D ligands and DR4 along with the downregulation of decoy receptor 1 (DcR1) in KG-1a cells [47]. A suppressor of cytokine signaling (SOCS3) was found to considerably impair TRAIL-driven apoptosis in cancer cells [48]. SOCS3 overexpression reduced apoptosis in DU145 cells co-treated with resveratrol and TRAIL. Moreover, SOCS3 inhibition increased apoptosis in TRAIL-treated LNCaP and PC-3 cells [48]. Survivin is a frequently overexpressed protein in TRAIL-resistant cancer cells. Resveratrol dose-dependently downregulated survivin in HepG2 cells. Moreover, 10 ng/mL TRAIL and 50 μmol/L resveratrol worked with effective synergy and increased the apoptotic rate up to 49.6% in HepG2 cells [49]. In xenografted tumors, resveratrol upregulated DR4, DR5, Bax, and p27(/KIP1) and inhibited the expression of cyclin D1 and Bcl-2 [50].

## 7. Regulation of STAT Signaling

**Figure 3:**
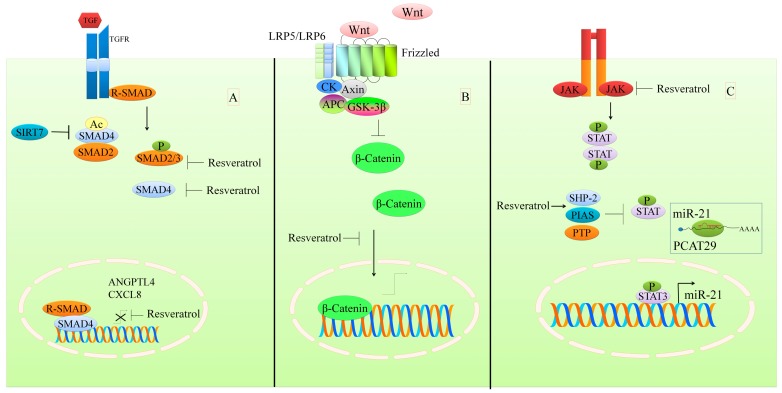
An overview of resveratrol-mediated targeting of multiple signaling pathways. (**A**) TGF/SMAD signaling pathway regulation by resveratrol; (**B**) WNT/Catenin pathway regulation by resveratrol; (**C**) Resveratrol increases negative regulators of STAT signaling (SHP-2, PIAS, and PTP) and inhibits JAK-mediated STAT phosphorylation. STAT3 transcriptionally upregulates miR-21, and targets the tumor suppressor gene PCAT29.

Prostate cancer associated transcript 29 (PCAT29), a tumor suppressor, is frequently overexpressed in prostate cancer and downregulated by interleukin (IL-6) in DU145 and LNCaP cells [51]. Significantly enhanced phopsphorylated-STAT3 has been observed following treatment with IL-6 (10 ng/mL) in DU145 and LNCaP cells [51]. STAT3 knockdown induced an increase in PCAT29 expression in both DU145 and LNCaP cells. Mechanistically, it was shown that STAT3 triggered the upregulation of miR-21, which consequently negatively regulated PCAT29 in PCa cells. Resveratrol repressed STAT signaling and stimulated expression of PCAT29 via repression of miR-21 [51] (Figure 3). Further, protein inhibitor of activated STAT3 (PIAS3) has been found to be downregulated in medulloblastomas, and resveratrol shown to significantly upregulate PIAS3 in UW228-2, UW228-3, and DAOY cells with the repression of p-STAT3 levels [52].

Resveratrol exerts inhibitory effects on the constitutive activation of STAT3 and STAT5. It inhibits the shuttling of STAT3 and STAT5 into the nucleus and significantly reduces the DNA binding affinity of STAT proteins in 786-O and Caki-1 cells [53]. Resveratrol was also found to markedly reduce the constitutive activation of Janus kinases (JAK1 and JAK2) in 786-O and Caki-1 cells. Protein tyrosine phosphatases (PTP and SHP-2) were considerably upregulated in resveratrol-treated 786-O and Caki-1 cells (Figure 3). Targeted inhibition of either PTP or SHP-2 substantially impaired the resveratrol-mediated decrease in the level of phosphorylated-STAT [53]. Resveratrol has also been shown to prevent the activation of JAK, resulting in the inhibition of the JAK-mediated phosphorylation of STAT1 [54].

## 8. Regulation of Other Signaling Pathways

### 8.1. AKT and MAPK

Resistance against different chemotherapeutic drugs is a frequently noted mechanism [55]. Docetaxel increased HER-2 in SK-BR-3 cells and induced Akt (Ser 473) phosphorylation within a time period as short as 30 min. Docetaxel induced extracellular signal-regulated kinase (ERK), c-Jun N-terminal kinase (JNK), and P38 phosphorylation, which was significantly inhibited by resveratrol [55]. It has also been noted that a high glucose-driven increase in the levels of phosphorylated-ERK and phosphorylated-p38 were inhibited by resveratrol in Panc-1 cells [56].

### 8.2. The ATM/p53 Pathway

ATM (ataxia telangiectasia mutated) is an important sensor of DNA damage and transduces the signals to downstream effectors, particularly p53 [57]. In glioblastoma-initiating cells treated with a combination of temozolomide and resveratrol, phosphorylated-ATM was found to be moderately upregulated, while p53 and phosphorylated-p53 were found to significantly increase. Resveratrol has also been found to be more effective against HCT-116 p53 wild-type colon carcinoma cells, as compared to HCT-116 p53^−/−^ cells [58]. Resveratrol induced DNA damage, as evidenced by the presence of multiple γ-H2AX foci after treatment with 25 μM resveratrol. DNA-damage-associated signaling was initiated through the activation of ATM kinase. p53 was found to be phosphorylated on serine 15 by ATM in response to resveratrol [58].

### 8.3. Multiple Signaling Pathways

Being a pleiotropic agent, resveratrol has been reported to target multiple proteins in ovarian cancer, markedly reducing NOTCH2 and HES1 in OVCAR-3 and CAOV-3 cells [59]. In CAOV-3 cells, resveratrol downregulated WNT2 and reduced the nuclear accumulation of β-catenin [59]. Moreover, the accumulation of STAT3 was evident in the nuclei of naïve OVCAR-3 cells, and resveratrol significantly reduced this nuclear accumulation. Although there is general agreement about the multi-protein targeting of resveratrol, there are still important mechanistic details related to the interwoven network of signal transduction pathways that remain to be elucidated.

## 9. Regulation of MicroRNAs by Resveratrol

**Figure 4:**
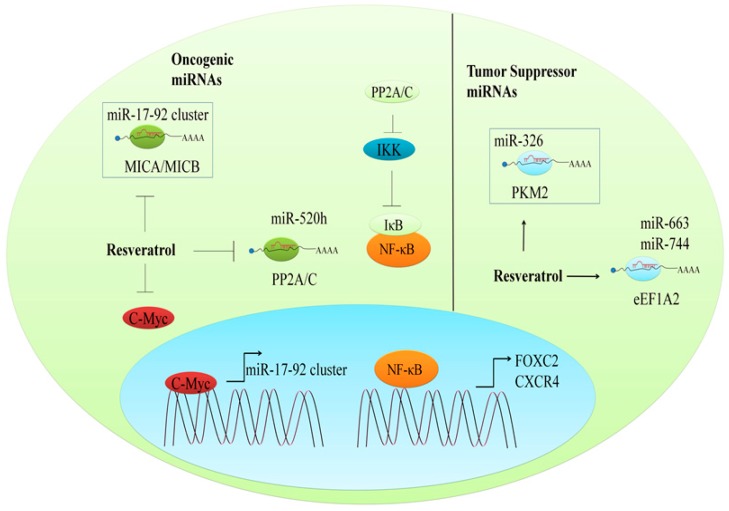
Resveratrol profoundly affects the miRNA machinery. It inhibits oncogenic as well as tumor suppressor miRNAs to affect the cellular oncogenic machinery, resulting in reduced expression of oncogenes and increased expression of tumor suppressor genes.

In just over two decades, since the discovery of the first microRNA (miRNA), there has been an exponential rise in the number of reports that provide in-depth analysis of regulation of signaling pathways by miRNAs in different cancers [60]. Categorically, miRNAs are classified into tumor suppressor and oncogenic miRNAs. Different strategies are currently being used to increase the expression of tumor suppressor miRNAs and downregulate oncogenic miRNAs. In this section, we will focus on the regulation of oncogenic and tumor suppressor miRNAs by resveratrol (Figure 4).

### 9.1. Regulation of Oncogenic miRNAs

Insulin-like growth factor binding protein-3 (IGFBP3) has been noted to be frequently downregulated in acute lymphocytic leukemia (ALL) cells [61]. Detailed mechanistic insights revealed that miR-1290 and miR-196b quantitatively controlled IGFBP3 in the ALL cell line. More importantly, resveratrol considerably reduced the expression of miRNA-196b/miRNA-1290 in TALL-104/SUP-B15 cell lines. The research findings were suggestive of a resveratrol-induced upregulation of IGFBP3 (mRNA, protein) in SUP-B15 as well as TALL-104 cells. Resveratrol arrested TALL-104 cells at the G1 phase and arrested SUP-B15 cells at the S phase. Resveratrol strongly induced apoptosis in the SUP-B15 and TALL-104 cells [61].

Activation of natural killer cells is a well-coordinated mechanism, triggered by signals derived from activating receptor ligation [62]. Natural killer group-2 member-D (NKG2D) or killer cell lectin-like receptor, subfamily-K, member-1 (KLRK1) is an extensively studied activating receptor of natural killer (NK) cells. In humans, the NKG2D ligand (NKG2DLs) family has eight glycoproteins present on the cell surface, including the major histocompatibility complex class I chain-related proteins A and B (MICA/B) [62]. Resveratrol was found to dose-dependently enhance MICA and MICB on the surface of breast cancer cells. Resveratrol-mediated increases in MICA and MICB were more pronounced in the MDA-MB-231 and BCap37 cell lines. Transfection of BCap37 cells with miRNA-17 inhibitors resulted in the upregulation of MICA and MICB, while transfection of BCap37 cells with miRNA-17 mimics resulted in the downregulation of MICA and MICB. c-Myc binding sites had previously been identified in transcriptional initiation sites in miR-17-92 clusters, and c-Myc triggered an increase in the expression of this gene cluster [62]. Resveratrol dose-dependently repressed c-Myc (mRNA, protein) in treated breast cancer cells, which consequently resulted in a reduction of miR-17. c-Myc knockdown resulted in a reduction of pri-miR-17-92 and miR-17, whereas c-Myc-overexpression markedly enhanced pri-miR-17-92 and miR-17 [62].

A chromatin immunoprecipitation assay revealed that resveratrol decreases the association of NF-κB with FOXC2 promoter [63]. Specific Akt inhibition resulted in the suppression of phosphorylated-Akt, FOXC2, and mobility of A549 cells. Resveratrol-mediated inhibitory effects on NF-κB activity were impaired in cancer cells with constitutively active Akt [63]. PP2A/C, a catalytic subunit of serine/threonine phosphatase PP2A, inhibited Akt activity by Akt dephosphorylation. Resveratrol, dose- and time-dependently, increased the expression and activity of PP2A/C protein in cancer cells. Resveratrol enhanced the levels of PP2A/C via the inhibition of miR-520h. Intriguingly, the resveratrol-mediated increase in PP2A/C was dramatically abolished after restoration of miR-520h expression [63]. For a deeper understanding of the crosstalk among these proteins, molecular analysis was conducted in cervical cancer cells, and the results suggested that the PP2A/C-driven dephosphorylation of the inhibitor of kappa-B kinase (IKK) resulted in its inactivation [64]. Inactivation of IKK protected IκB from phosphorylation by IKK; consequently, activation of NF-κB was inhibited. Inactivation of NF-κB resulted in transcriptional repression of its target gene, CXCR4 [64]. These findings provide insight into the mechanisms by which miR-520h quantitatively reduces PP2A/C to facilitate the tumor-promoting activities of NF-κB via the transcriptional upregulation of its target genes.

### 9.2. The Regulation of Tumor Suppressor miRNAs

Pyruvate kinase M2 (PKM2) has been found to be overexpressed in different cancers [65]. Resveratrol, dose-dependently, increased the mRNA and protein expression levels of GRP78, CHOP, and caspase-12 in both HeLa and DLD1 cells. A reversal of these changes was observed in PKM2-overexpressing cancer cells. Mechanistically, it was shown that resveratrol repressed PKM2 by increasing the expression of miR-326. Interestingly, resveratrol-mediated repressive effects on PKM2 were impaired in miR-326 inhibitor-transfected cancer cells [65]. 

Stem cell factor (SCF, KITLG) is frequently overexpressed in colorectal cancer cells [66]. KITLG is quantitatively controlled by miR-34c. Studies have shown that p53 transcriptionally upregulated miR-34c in colorectal cancer tissues and cell lines. Resveratrol was noted to increase p53 in p53-positive HT-29 cells, and miR-34c upregulation was more pronounced in p53-positive HT-29 cells, as compared to p53-negative HCT-116 cells [66]. Overexpression of Isoform A2 of eukaryotic translation elongation factor (eEF1A2) played a contributory role in breast cancer progression [67]. miR-663 and miR-744 have been found to negatively regulate eEF1A2 in breast cancer. Resveratrol induced a 4.5-fold upregulation of miR-663 and a two-fold increase in miR-744. It also reduced EEF1A2 in breast cancer cells. A resveratrol-induced decrease in EEF1A2 levels was not found in MCF7 cells transfected with inhibitors of miR-663 and miR-744 [67]. Resveratrol was noted to reduce MMP2 via upregulation of miR-328 in osteosarcoma cells [68]. The resveratrol-mediated suppressive effects on MMP2 were impaired in HOS cells transfected with miR-328 inhibitors, whereas transient or stable overexpression of miR-328 markedly reduced the invasive potential of HOS cells [68].

## 10. Resveratrol in Clinical Trials

MPX, powdered muscadine grape skin that contains ellagic acid, resveratrol, and quercetin, was tested for efficacy in biochemically recurrent prostate cancer patients [69]. 6 out of 14 patients dropped out of the ongoing trial because the disease progressed post-treatment for a median of 15 months. Seven patients remained in study. More importantly, the lack of dose-limiting toxicities was encouraging even at a high dose of 4000 mg/day [69].

Randomized double blind placebo control trials have gained appreciation as the “gold standard” of epidemiological studies. Recently, a trial was conducted in which 22 biochemically recurrent prostate cancer patients were enrolled [70]. These patients had a moderate rise rate. Doubling time of prostate specific antigen (PSA) was 4–15 months with no evidence of metastasis. A non-significant rise in the log-slope of PSA was experienced by the treatment group. Pre-treatment doubling time was 10.2 months, whereas post-treatment doubling time was 5.5 months. However, no change in the log-slope of PSA was experienced by the placebo group. Doubling time before treatment was 10.8 months, while the doubling time after treatment was 10.9 months [70].

A Phase I clinical trial was conducted where colon cancer patients were enrolled in a study designed to evaluate the effects of low doses of resveratrol-containing freeze-dried grape powder (GP) and a plant-derived resveratrol formulation on the Wnt signal transduction cascade in the colon [71]. Patients treated with resveratrol/GP did not show any change in the Wnt target gene expression [71]. In another study, SRT501, micronized resveratrol, was given 5.0 g daily for 2 weeks to colorectal cancer patients. SRT501 was also given to patients with liver metastases scheduled to undergo hepatectomy [72]. The mean plasma level of resveratrol after single administrations of SRT501 was 1942 ± 1422 ng/mL. Resveratrol was detected in hepatic tissues after the administration of SRT501 (up to 2287 ng/g) [72]. The promising pre-clinical data has led to the interest in clinical evaluation of resveratrol. More elaborate trials need to be conducted to further establish the clinical relevance of resveratrol’s anticancer properties.

## 11. Concluding Remarks

Deregulation of spatio-temporally controlled cell signaling pathways contributes to cancer initiation, progression, and its metastatic spread. Therefore, the identification of novel anticancer therapeutics, such as natural products with significant anticancer activity and an ability to target multiple proteins of oncogenic pathways, is required. Resveratrol has been shown to modulate key regulators of oncogenic signaling pathways. It strongly inhibits the TGF/SMAD pathway and reduces phosphorylated SMADs, resulting in the inhibition of cancer cell proliferation and metastasis. Resveratrol has shown experimentally verified activity against WNT signaling and can inhibit the translocation of β-catenin into the nucleus. Resveratrol, context-dependently, regulates the NOTCH pathway in cancer cells, all of which underlines its flexible and potent anticancer activity. Reduced apoptosis is a hallmark of aggressive cancer cells, and resveratrol re-balances pro- and anti-apoptotic proteins to improve the efficacy of TRAIL-based therapeutics. It seems exciting to note that resveratrol may prove to be a clinically effective agent; however, lower bioavailability is a major shortcoming that needs to be overcome. In accordance with this idea, nanotechnological strategies are currently being tested to enhance the bioavailability of resveratrol. Resveratrol has been shown to efficiently upregulate various tumor suppressor miRNAs in different cancers, and its suppressive effects on oncogenic miRNAs have also been well documented. Keeping in view the multi-targeted approach and its robust anticancer effects involving multiple signaling pathways and molecular targets, resveratrol has enormous potential to be considered as an important and pharmacologically effective agent in the fight against cancer.
